# Editorial: Recent advancements in brain-computer interfaces-based limb rehabilitation

**DOI:** 10.3389/fnhum.2024.1466450

**Published:** 2024-08-21

**Authors:** Kishor Lakshminarayanan, Deepa Madathil, Bhaskar Mohan Murari, Rakshit Shah

**Affiliations:** ^1^Department of Sensors and Biomedical Tech, School of Electronics Engineering, Vellore Institute of Technology, Vellore, Tamil Nadu, India; ^2^Jindal Institute of Behavioural Sciences, O. P. Jindal Global University, Sonipat, Haryana, India; ^3^Department of Orthopaedic Surgery, University of Arizona, Tucson, AZ, United States

**Keywords:** brain-computer interface (BCI), wheelchair, music therapy, virtual reality, robot

## Introduction

Recent advancements in rehabilitation and brain-computer interface (BCI) technology have led to significant improvements in understanding and enhancing human performance, particularly for individuals with physical and cognitive impairments. This editorial highlight four notable studies published in early 2024 that showcase innovative approaches in community mobility, immersive virtual reality (VR), music therapy, and EEG-based movement classification. These studies collectively demonstrate the potential of integrating advanced technologies to improve rehabilitation outcomes and quality of life.

Fasipe et al. reviewed current methods and technologies for measuring community mobility and participation (CMP) among manual wheelchair users (MWU). Through a comprehensive review of articles from 2017 to 2023, the researchers identified three primary methods for assessing CMP: self-surveys, clinical measurements, and remote measurements. Self-surveys included tools like the Life Space Assessment and the Wheelchair Use Confidence Scale for Community Mobility, clinical measurements included the 6-Minutes Push Test and VO2 Max Test, and remote measurements used GPS or accelerometer data.

The review article also highlighted factors affecting CMP, including community factors, environmental barriers, physical fitness, and social support. Rehabilitation methods to improve CMP, such as brain-computer interface (BCI) training, neuro-rehabilitation techniques involving VR and EEG, and community-based programs, were discussed. The study concluded that while current methods for measuring CMP were beneficial, they were limited and needed improvement. Future research should focus on developing more accurate and comprehensive methods for measuring CMP and identifying effective rehabilitation techniques to enhance it.

Degirmenci et al. conducted a study targeting the classification of finger movements and the brain's idle state using EEG signals analyzed through intrinsic time-scale decomposition (ITD) and proper rotational components (PRCs). The study evaluated both subject-independent and subject-dependent cases, showing high performance in classifier accuracy using ANOVA for feature selection.

EEG data from eight subjects were decomposed, and features were extracted using ITD, focusing on low-frequency signals from the same cortical region. Eight machine learning algorithms were employed, with ensemble learning achieving the highest accuracy of 55%. The study's design included EEG data acquisition, ITD-based feature extraction, feature reduction, classification, and performance evaluation. The results highlighted the potential of combining PRCs and ITD to classify finger movements and brain states, marking a significant advancement in EEG-based movement classification.

Larsen et al. developed a method for synchronizing EEG and eye tracking in a fully immersive VR environment, presenting significant implications for neuroscience research. The study implemented a hybrid steady-state visually evoked potential (SSVEP) based BCI speller within VR, using research-grade eye-tracking devices integrated with high-accuracy EEG recording.

The experimental setup involved two computing devices: one desktop computer running Unity and Neurotype and one laptop running EEG recorder. Data transmission occurred at 500 Hz for EEG and 250 Hz for eye movement data, with 661 blinks recorded from four participants. The study focused on calculating the latency and jitter of the VR-integrated eye tracker with the SSVEP speller, achieving high temporal accuracy with an average latency of 0.1 milliseconds and mean jitter of 0.2 milliseconds. The findings demonstrated the feasibility of combining commercial EEG and VR technologies for recording and analyzing brain activity in response to visual stimuli, highlighting the potential for advanced BCI applications in immersive environments.

Liu et al. proposed a study protocol to explore the benefits of combining reminiscent music therapy with robot-assisted rehabilitation for older stroke patients with upper limb dysfunction. The protocol outlined a single-blind, three-arm randomized controlled trial with three types of rehabilitation treatments: usual rehabilitation care, robot-assisted rehabilitation, and robot-assisted rehabilitation with reminiscent music therapy.

Participants receiving reminiscent music therapy showed improved attention, reduced fatigue, and enhanced physical wellbeing due to the positive effects of musical sound and rhythm. The study used a three-degree freedom rehabilitation robot with five training modes and a music library of 100 songs, divided into ten sets. Outcomes were measured using various scales, including the self-esteem scale, stroke-self efficacy questionnaire, positive and negative effect scale, Fugl-Meyer test, and modified Barthel index. Statistical analyses, including paired sample *t*-tests and chi-square tests, were planned to determine the significance of the results. This protocol aimed to demonstrate the novel benefits of combining music therapy with robot-assisted rehabilitation.

## Conclusion

The recent advancements in BCI-based limb rehabilitation, exemplified by these studies, have paved the way for innovative approaches to enhance human performance and rehabilitation outcomes. The integration of technologies such as VR, EEG, BCI, and music therapy holds great promise for improving the quality of life for individuals with physical and cognitive impairments. As research continues to evolve, it is crucial for practitioners, clinicians, and educators to embrace these advancements and explore new possibilities for enhancing rehabilitation and human performance.

